# Is It Time to Begin a Public Campaign Concerning Frailty and Pre-frailty? A Review Article

**DOI:** 10.3389/fphys.2017.00484

**Published:** 2017-07-11

**Authors:** Jerzy Sacha, Magdalena Sacha, Jacek Soboń, Zbigniew Borysiuk, Piotr Feusette

**Affiliations:** ^1^Faculty of Physical Education and Physiotherapy, Opole University of Technology Opole, Poland; ^2^Department of Cardiology, University Hospital of the University of Opole Opole, Poland; ^3^Outpatient Cardiology Clinic Nysa, Poland

**Keywords:** frailty, pre-frailty, cognitive frailty, exhaustion, weakness, weight loss, sarcopenia, low physical activity

## Abstract

Frailty is a state that encompasses losses in physical, psychological or social domains. Therefore, frail people demonstrate a reduced potential to manage external stressors and to respond to life incidents. Consequently, such persons are prone to various adverse consequences such as falls, cognitive decline, infections, hospitalization, disability, institutionalization, and death. Pre-frailty is a condition predisposing and usually preceding the frailty state. Early detection of frailty (i.e., pre-frailty) may present an opportunity to introduce effective management to improve outcomes. Exercise training appears to be the basis of such management in addition to periodic monitoring of food intake and body weight. However, various nutritional supplements and other probable interventions, such as treatment with vitamin D or androgen, require further investigation. Notably, many societies are not conscious of frailty as a health problem. In fact, people generally do not realize that they can change this unfavorable trajectory to senility. As populations age, it is reasonable to begin treating frailty similarly to other population-affecting disorders (e.g., obesity, diabetes or cardiovascular diseases) and implement appropriate preventative measures. Social campaigns should inform societies about age-related frailty and pre-frailty and suggest appropriate lifestyles to avoid or delay these conditions. In this article, we review current information concerning therapeutic interventions in frailty and pre-frailty and discuss whether a greater public awareness of such conditions and some preventative and therapeutic measures may decrease their prevalence.

## Introduction

Frailty is a geriatric syndrome caused by a multisystem decrease in reserve capability and is associated with a high risk for various adverse outcomes. “Frailty is not synonymous with either comorbidity or disability, but comorbidity is a risk factor for frailty; however disability is an outcome of frailty”—pre-frailty is a condition predisposing and directly preceding frailty (Buchner and Wagner, 1992; Fried et al., 2001). In fact, the frailty state is associated with a variety of adverse consequences, such as falls, cognitive decline, infections, hospitalization, disability, institutionalization and death (Fried et al., 2001; Abellan van Kan et al., 2008; Pilotto et al., 2012). Frail patients present much worse prognoses than non-frail patients, particularly in cardiovascular diseases (Singh et al., 2014). Moreover, frailty impairs the effects of invasive treatments in these disorders, e.g., percutaneous coronary interventions, transcatheter aortic valve implantations or coronary artery bypass grafting (Singh et al., 2011, 2014; Green et al., 2012). Frailty also imposes a significant financial burden on health systems, particularly because frailty appears to have an incremental effect on ambulatory health expenditures (Sirven and Rapp, 2017). Awareness of these facts may afford us an opportunity to develop cost-effective care for this group of people, resulting in improvement in long-term care and its outcomes (Wyrko, 2015). However, despite numerous studies addressing this condition in recent years, frailty as an entity is not commonly recognized in the general population or even by some medical societies, and there are no consistent preventative and therapeutic strategies dedicated to this disorder. Because population aging is associated with a higher prevalence of frailty and pre-frailty (Fernández-Garrido et al., 2014; Sergi et al., 2015), it is necessary to familiarize societies with these states. Moreover, if we want to improve the quality of life of elderly persons and reduce expenses for their care in the future, we should take preventative measures against frailty now (Wyrko, 2015). Therefore, it is time to begin treating frailty like other population-affecting diseases such as obesity, diabetes or hypertension. Appropriate prospective studies are needed to define which preventative lifestyle interventions should be implemented to ensure good physical and mental conditions in senility. Social campaigns could draw societies' attention to proper life habits that may be effective to avoid not only diabetes and cardiovascular diseases but also age-related frailty. In this article, we review up-to-date information concerning therapeutic interventions in frailty and pre-frailty and discuss whether a greater public awareness of these entities and some preventative and therapeutic measures may reduce their prevalence.

## Primary diagnostic methods of frailty and pre-frailty

**Figure 1:**
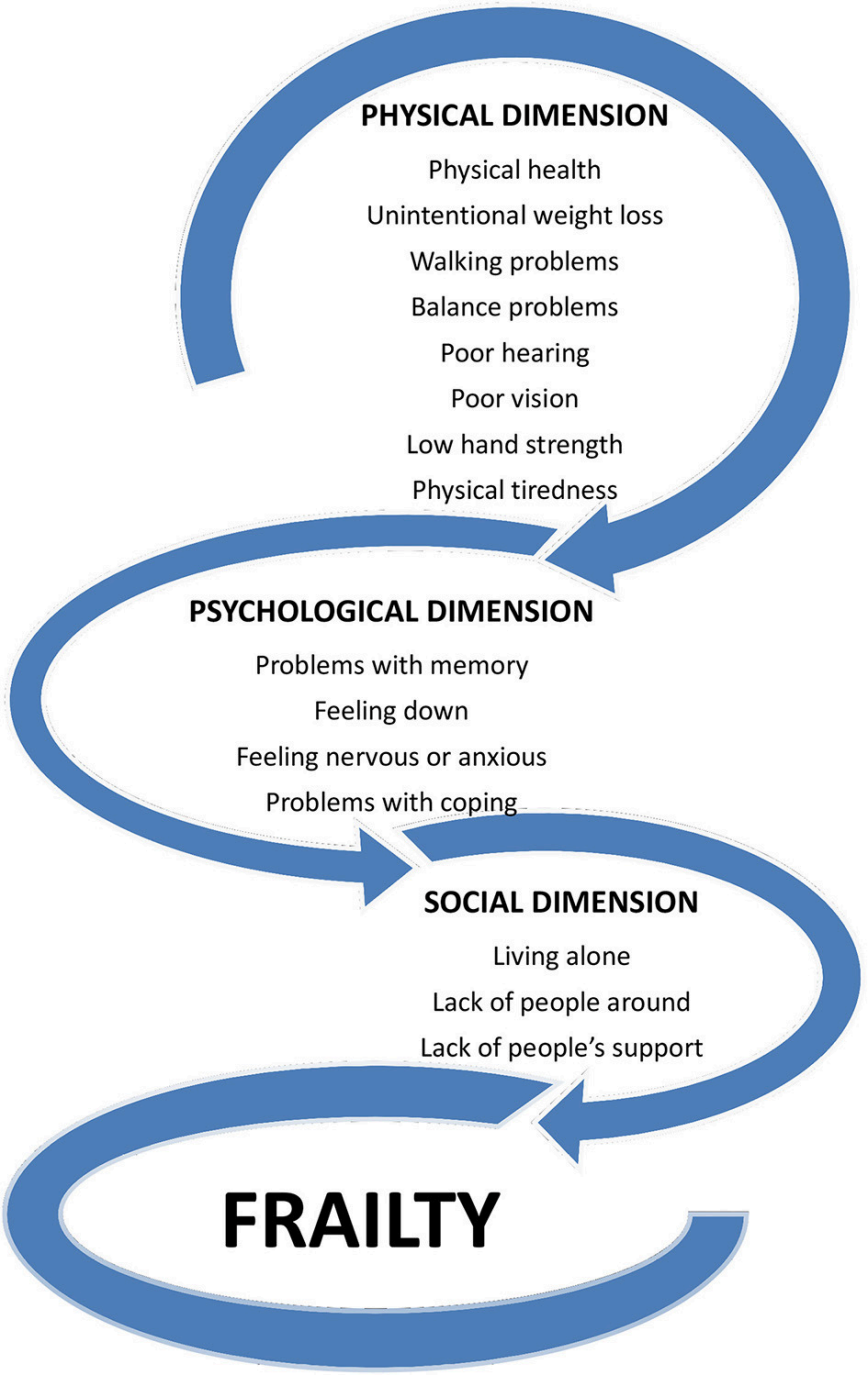
Multidimensional concept of frailty integrating various domains of human functioning that, by interaction, may accelerate frailty development (the diagram reflects the structure of the Tilburg Frailty Indicator by Gobbens et al., 2010b).

Frailty is a dynamic condition that includes losses in physical, psychological and social fields; frail people thus present a reduced capability to manage external stressors and to respond to life incidents (Buchner and Wagner, 1992; Fried et al., 2001). Until now, numerous frailty diagnostic tools have been proposed; in one recent systematic review, the authors identified 67 various frailty instruments (Buta et al., 2016). The abundance of such instruments suggests that no agreement on an operational definition of frailty has yet been reached. Indeed, frailty is diagnosed with various tools, which in general can be grouped into two types of conceptualisations: unidimensional, based on the physical and biological dimension, and multidimensional, based on the connections between physical, psychological, and social domains (Figure 1; Fried et al., 2001; Markle-Reid and Browne, 2003; Carrière et al., 2005; Abellan van Kan et al., 2008, 2010; Gobbens et al., 2010b; De Vries et al., 2011; Roppolo et al., 2015).

On the unidimensional level, the best-known conceptualisation of frailty is that proposed by Fried et al. (2001), which formed the basis for the Cardiovascular Health Study Index (CHS), also known as the Physical Frailty Phenotype (PFP). According to this method, frailty should be defined by five features: unintentional weight loss, self-reported exhaustion, muscle weakness, slow walking speed and low physical activity, with the presence of 3 or more of these features denoting frailty, 1 or 2 denoting pre-frailty, and none denoting no frailty (Fried et al., 2001). Consistent with this type of conceptualisation, age-related sarcopenia must be a key element of frailty but is not synonymous with frailty. By definition, age-related sarcopenia is low muscle mass and low muscle strength or physical performance when no other cause except aging is evident (Cruz-Jentoft et al., 2010; Bosaeus and Rothenberg, 2016). In fact, frailty and sarcopenia overlap one another; a majority of frail older persons present sarcopenia, although not all older people with sarcopenia are frail. However, the concept of frailty, i.e., a general vulnerability to various external stressors, extends far beyond a physical dimension; a multidimensional conceptualisation of frailty therefore reflects a wider context of human functioning (Bauer and Sieber, 2008; Cruz-Jentoft et al., 2010; Bosaeus and Rothenberg, 2016).

The multidimensional approach to frailty is based on an analysis of interactions among various domains: physical, psychological and social (Walston et al., 2006; Abellan van Kan et al., 2008, 2010; Gobbens et al., 2010b). In this context, frailty is considered a state affecting people who present losses in one or more of these levels of human functioning (Mitnitski et al., 2001; Rockwood et al., 2005, 2007; Searle et al., 2008; Gobbens et al., 2010a). Such a definition was operationalized into various tools used to assess frailty (De Vries et al., 2011; Sternberg et al., 2011; Roppolo et al., 2015). One of those tools is the frailty Index (also known as the Deficit Accumulation Index, DAI), developed as a sum of “deficits in health,” i.e., symptoms, illnesses, physical, and cognitive impairments, disabilities, psychosocial risk factors, and laboratory abnormalities: more deficits identified in a given person indicate a greater likelihood that a person is frail (Mitnitski et al., 2001; Rockwood et al., 2005, 2007). The frailty index is generally presented as a ratio of deficits identified in relation to the number of all deficits considered. For instance, if 50 deficits are considered and 10 are identified, the individual's frailty index is 10/50 = 0.2 (Mitnitski et al., 2001; Searle et al., 2008). The frailty index appears to be consistent across different studies, although not every frailty index considers similar deficits or the same number of deficits (Mitnitski et al., 2001; Goggins et al., 2005; Rockwood and Mitnitski, 2007; Rockwood et al., 2007; Searle et al., 2008). This index is significantly associated with adverse outcomes such as deterioration in health status, risk of institutionalization and death (Rockwood et al., 2007). However, the greatest concern regarding this approach is that the frailty index is based on the accumulation of a wide range of different deficits and consequently must include disability as a component. The index scarcely differentiates between people at risk (i.e., frail) and those already disabled (Abellan van Kan et al., 2010).

One of the recent and quite promising multidimensional instruments, based on a structural questionnaire, is the Tilburg Frailty Indicator (TFI) (Gobbens et al., 2010b; see Figure 1). The TFI consists of two parts: Part A comprises 10 questions on frailty determinants (e.g., age, gender, marital status, education level, and way of life); Part B comprises 15 frailty elements arranged according to three different aspects. The physical aspect (0–8 points) consists of eight items related to physical health, unintentional weight loss, difficulty walking, and problems with balance, hearing, vision, hand strength and physical tiredness. The psychological aspect (0–4 points) comprises four components related to cognition, depression, anxiety, and coping. The social aspect (0–3 points) consists of three elements associated with living alone, social relations and social support. The tool's total score may rank from 0 to 15–by definition, frailty is established if the total TFI score is at least 5 (Gobbens et al., 2010b).

Studies comparing uni- and multidimensional measures of frailty have demonstrated large differences in their ability to identify frail older adults (Hoogendijk et al., 2013; Theou et al., 2013; Jung et al., 2014; Roppolo et al., 2015). For example, in one study, the CHS revealed the highest association with the physical domain of the TFI, whereas the correlations with the psychological and social domains were much weaker, although significant (Roppolo et al., 2015). Importantly, both instruments presented good correlation with disability; however, the CHS demonstrated low sensitivity and high specificity, whereas the TFI exhibited high sensitivity and low specificity. Because frailty is a preclinical state, it seems better to prefer high sensitivity over high specificity, rendering it possible to identify persons at risk who may benefit from preventive management (Roppolo et al., 2015). However, it should be noted that the CHS with its pre-frailty state may also be a sensitive measure to detect an early reduction in reserve capacity in apparently healthy elderly men and women (Fried et al., 2001).

Early detection of any form of functional degradation in elderly individuals appears to be critical if one intends to introduce interventions to preserve their biological, psychological and social status. Therefore, if frailty instruments include disability, functional decline and cognitive impairment as components of frailty, these instruments in fact identify people who are already disabled (Brody et al., 1997, 2002; Rockwood et al., 1999, 2005; Saliba et al., 2001; Mitnitski et al., 2002a,b; Carrière et al., 2005; Ravaglia et al., 2008; Sarkisian et al., 2008; Avila-Funes et al., 2009). Hence, potential interventions in such groups would resemble secondary prevention trials (Sternberg et al., 2011). However, if frailty definitions consider disability, functional decline and dementia as outcomes of frailty (Chin A Paw et al., 1999; Fried et al., 2001; Saliba et al., 2001; Carrière et al., 2005; Puts et al., 2005b; Ensrud et al., 2008; Ravaglia et al., 2008; Gobbens et al., 2010b), they allow identification of populations for whom a management plan would be analogous to primary prevention therapy (Sternberg et al., 2011).

As the transition from disability to robust status is extremely difficult, it is reasonable to begin addressing the problems of the elderly in the early preclinical stages (i.e., pre-frailty and frailty) to stop or delay functional deteriorations. Therefore, in the selection of a frailty instrument, it is crucial to consider the intended purpose, clinical context and information regarding how the instrument has been used in the past (Buta et al., 2016).

## Pre-frailty state

It has been suggested that the recognition of pre-frailty may deliver an opportunity to introduce effective management that may improve outcomes in terms of hospitalization, disability and mortality (Gary, 2012; Von Haehling et al., 2013). The incidence of pre-frailty in the elderly aged over 65 years ranges between 35 and 50% and is much higher than the prevalence of frailty, which ranges from 7 to 12% in subjects aged 65 years or more and approximately 25% in those older than 85 years (Fernández-Garrido et al., 2014; Sergi et al., 2015).

Pre-frailty is recognized when one or two criteria of the CHS are met (Fried et al., 2001). The prevalence of such criteria in pre-frail individuals differs according to various authors. In one study, the prevalence of these components was found to be 40.5% for muscle weakness, 29.0% for unintentional weight loss, 17.1% for low physical activity, 11.5% for self-reported exhaustion, and 2.0% for slow walking speed (Danon-Hersch et al., 2012); however, in another study, weakness was the most common initial manifestation in pre-frail women and low activity and slowness were the second and third most reported (Xue et al., 2008). Notably, exhaustion and unintentional weight loss seldom develop alone but generally occur in conjunction with other reductions in compensatory mechanisms (Bortz, 2002; Kamel, 2003; Drey et al., 2011). Individuals with exhaustion have higher scores on the geriatric depression scale, suggesting a correlation between these two conditions (Drey et al., 2011). In general, weight loss is relatively rarely observed in pre-frail people and appears to be associated with the end stage of frailty, which is strongly associated with mortality (Fernández-Garrido et al., 2014).

Similar to frailty, pre-frailty is more common in women than in men and is associated with lower levels of education and socio-economic status (Raji et al., 2010; Danon-Hersch et al., 2012). In fact, women accumulate more deficits than men of the same age, and in both genders, this accumulation is associated with mortality, although men exhibit a higher risk of mortality (Mitnitski et al., 2002b; Puts et al., 2005a). There is a significantly higher level of comorbidities in pre-frail than in non-frail individuals, with a particularly higher frequency of chronic cardiovascular and respiratory diseases, diabetes mellitus, osteoporosis, arthritis, balance problems, depression and cognitive impairment (Raji et al., 2010; Danon-Hersch et al., 2012; Fernández-Garrido et al., 2014).

Pre-frailty is associated with a number of risk factors, such as higher hemoglobin A_1c_, inflammation markers, uric acid and waist circumference as well as lower vitamin D levels and ankle brachial index and some alterations in hormone levels (i.e., testosterone, dehydroepiandrosterone [DHEA] and its sulfate ester [DHEAS], parathyroid hormone and cortisol) (Cappola et al., 2009; Shardell et al., 2009; O'Connell et al., 2011b; Smit et al., 2012; Fernández-Garrido et al., 2014; Johar et al., 2014; Sergi et al., 2015). Low testosterone levels are independently associated with pre-frailty and frailty in both elderly men and women (Wu et al., 2010). In general, women with pre-frailty and frailty have lower levels of anabolic hormones (testosterone, DHEAS and insulin-like growth factor 1 [IGF-1]) than their non-frail counterparts at similar ages—moreover, the absolute burden of all anabolic hormonal deficiencies better predicts frailty status than a single hormonal deficiency (Cappola et al., 2009). The probability of frailty may reveal a U-shaped relation with women's free testosterone levels: lower and higher levels are associated with the chance of being frail in women, whereas in men, this probability is linearly and inversely related to testosterone levels. Moreover, obesity modifies the effects of testosterone on frailty in females but not in males (Carcaillon et al., 2012).

The accumulation of these subclinical conditions may appear as pre-frailty states, which, in addition to other comorbidities and older age, may increase the risk of (particularly) cardiovascular diseases (Flint, 2015). Notably, pre-frailty is particularly associated with heart failure events, and indeed, heart failure is the most frequent mode of cardiovascular diseases among pre-frail and frail people (Newman et al., 2001, 2006; Woods et al., 2005; Sergi et al., 2015). However, some data suggest that the reverse scenario between pre-frailty and cardiovascular illnesses may also be true; clinical and subclinical cardiovascular disorders may increase the risk of pre-frailty (and frailty), therefore postulating a bidirectional relation between these two entities (Newman et al., 2001; Gale et al., 2014; Ricci et al., 2014; Flint, 2015; Sergi et al., 2015). This bidirectional causal relationship enables considering the pre-frailty state and cardiovascular diseases as a vicious cycle in which one feeds the development of the other (Flint, 2015). Interventions directed at breaking this vicious cycle should be effective in the early disease stage, i.e., in patients with pre-frailty or preclinical cardiovascular illnesses, and management should focus on both the reduction of cardiovascular risk factors and the augmentation of overall physiologic reserves (Flint, 2015).

## Physical, nutritional and medical interventions on frailty

With increasing age, the progressive decrease in voluntary physical activity leads to a higher risk of frailty; regular exercise should therefore be beneficial to older people who are frail or at high risk of frailty, i.e., pre-frail (Walston et al., 2006). The rationale to intervene with physically frail elderly people comes from the concept that the pathway toward frailty relies on the mechanism in which sarcopenia (beyond inflammation and neuroendocrine deregulation) plays a primary role; hence, physical activity interventions should be a key treatment element in physical frailty independent of concomitant diseases (Woods et al., 2005).

Recent systematic reviews and meta-analysis indicate that exercise interventions improve gait speed and physical function measured by physical performance scales in frail elderly subjects. However, the results are inconclusive for endurance outcomes, and no consistent effect was observed for balance and functional status. Moreover, many uncertainties exist with regard to which exercise characteristics (i.e., type, frequency and duration) are the most effective in elderly populations (Giné-Garriga et al., 2014; de Labra et al., 2015). Another meta-analysis demonstrated that compared with the control group, the exercise group increased their gait speed and balance function and improved their performance in the activities of daily living; nevertheless, no significant benefit in the group's quality of life was observed (Chou et al., 2012). In their meta-analysis, de Vries et al. (2012) demonstrated that a physical exercise therapy positively affects mobility and physical functioning; moreover, high-intensity exercise interventions appear to be somewhat more effective in improving physical functioning than low-intensity exercises. However, the effect on physical activity and quality of life is not clear, and no ultimate conclusions on the most efficient type of physical exercise therapy can be reached (de Vries et al., 2012). In their systematic review, Cadore et al. (2013) revealed that multicomponent exercise intervention with balance, strength and endurance training is the best strategy to decrease the rate of falls and improve gait, balance and strength in physically frail older individuals. Systematic reviews by Daniels et al. (2008) and Theou et al. (2011) also provided arguments that multicomponent exercise training may have the best positive effect on physical performance; hence, such an approach should be commonly used for the management of frail older adults.

Although a multicomponent approach seems to be the most effective strategy with which to slow the frailty progress, resistance training appears to be a key element in preventing sarcopenia and falls, and maintaining functional capacity (Cadore et al., 2014; Bosaeus and Rothenberg, 2016). Reduced muscle mass and strength are fundamental components of frailty; however, aging muscles can positively respond to increased activity, particularly to resistance exercises (Fiatarone et al., 1994). This type of training ultimately has the largest effect on improving and preserving lean muscle mass (Cruz-Jentoft et al., 2014; Bosaeus and Rothenberg, 2016). Meta-analyses by Liu and Latham (2009) demonstrated that progressive resistance training improves physical functioning in older people in the areas of muscle strength and the performance of certain simple and complex activities. Although serious adverse events of such activity are rare, caution should be taken when implementing this approach with clinical populations at higher risk of injury (Liu and Latham, 2009).

Despite all of these reports documenting that older adults may benefit from regular exercise training, more trials are required to determine the most appropriate exercise programme, i.e., the type, intensity, frequency and duration of training (Chin A Paw et al., 2008).

Another primary risk factor for the onset of frailty is malnutrition, which is indeed quite prevalent in geriatric populations. Malnutrition significantly influences the development of frailty because weight loss leads to weakness, exhaustion, slow walking speed and low physical activity—hence, nutritional screening is highly important in frail individuals (Lilamand et al., 2015; Artaza-Artabe et al., 2016). Hypoalbuminemia is a marker of malnutrition associated with a loss of muscle mass and is also a prognostic factor for mortality in the elderly population (Cabrerizo et al., 2015). A diet with a high protein content may have a beneficial effect on health, help in recovery from diseases and maintain functional status in those persons (Bauer et al., 2013; Volpi et al., 2013). Some studies suggest that the amount of protein intake in elderly patients, which is necessary to stimulate protein synthesis, should range between 1.2 and 1.5 g/kg/day (Gaffney-Stomberg et al., 2009; Mithal et al., 2013). Indeed, nutritional supplementation for frail people has been demonstrated to slow their functional decline, as measured by the Short-Physical Performance Battery and tests of hand grip strength (Kim and Lee, 2013). In addition, the combination of resistance exercise and protein supplementation after exercise may be even more effective in increasing muscle mass, strength, and physical ability in elderly people (Artaza-Artabe et al., 2016). Not all studies addressing dietary interventions, however, provide uniform results: there is also relatively low adherence to the nutritional treatment over a long period of time among older people, preventing a consistent consensus on the effects of nutritional supplementation in frailty (Artaza-Artabe et al., 2016).

The etiology of sarcopenia in frail people involves multiple mechanisms, including the age–related decline in the level of anabolic hormones (Fried et al., 2005; O'Connell et al., 2011b). In this context, testosterone, the primary androgenic hormone in men, appears to play a critical role because it has a strong anabolic influence on skeletal muscle (Bhasin et al., 2005). Studies have demonstrated that a low bioavailable testosterone level is associated with frailty not only in men but also, to some extent, in women (Cawthon et al., 2009; Wu et al., 2010; Carcaillon et al., 2012). Treatment with testosterone, particularly at higher doses, may improve body composition and modestly increase muscle strength in older men (Bhasin et al., 1996; O'Connell and Wu, 2014). Although a response to this type of therapy is not generally reflected in physical functioning such as gait speed, mobility or daily living activities, improvement can be observed in muscle strength, i.e., grip and lower limb strength (O'Connell and Wu, 2014). It is possible that testosterone alone may be relatively ineffective in ameliorating physical functioning and should instead be combined with different exercise trainings to create a broad functional benefit (Bhasin, 2010). An overall improvement in strength and lean body mass typically appears within 6 months and lasts for the duration of testosterone treatment (Snyder et al., 1999; Wittert et al., 2003; Page et al., 2005). Unfortunately, the majority of these benefits are lost 3–6 months after ending the therapy (O'Connell et al., 2011a; Sattler et al., 2011). Nevertheless, a common use of testosterone in older men is restricted by concerns regarding cardiovascular and prostatic adverse effects (O'Connell and Wu, 2014). In one of the trials, testosterone therapy was discontinued because of an excess of cardiovascular events (Basaria et al., 2010), and the meta–analysis by Calof et al. (2005) suggested that testosterone treatment doubles the rate of prostate events. Thus, large-scale, long duration, appropriately powered clinical studies are necessary to establish the safety of this type of therapy before it can be approved for the management of frail, or even pre-frail, older male populations.

IGF-1 is another anabolic hormone that plays a role in the preservation of muscle mass and strength and may prevent apoptosis and protect from oxidative stress (Arvat et al., 2000). Because of these features, IGF-1 may be considered a key element of nutritional, hormonal and inflammatory hypotheses of frailty (Maggio et al., 2013). Notably, some minerals, such as magnesium, selenium, and zinc have some beneficial effects on IGF-1 levels (i.e., tissue production or liver secretion) and muscle function, providing hope for a potential improvement in physical performance among elderly patients (Maggio et al., 2013). However, the real effectiveness of this and other probable therapeutic interventions (e.g., antioxidants, anti-inflammatory agents, nutritional supplements) appears to be limited and thus far has not been sufficiently explored in detail (Laosa et al., 2014). Therefore, more clinical data are imperative before proposing such curative options to frail people.

In this context, a condition that has been more deeply investigated and may potentially contribute to a sedentary lifestyle and immobility and therefore to frailty is vitamin D deficiency. Low levels of 25(OH)D are quite common in elderly people (particularly those institutionalized) (Visser et al., 2006) and have been demonstrated to be associated with balance problems, falls, fractures and pain (Lips, 2001; Zamboni et al., 2002; Mascarenhas and Mobarhan, 2004; Snijder et al., 2006). Some convincing clinical data (including meta-analysis of randomized trials) indicate that vitamin D supplementation can reduce falls and improve muscle strength and walking distance in older people (Verhaar et al., 2000; Montero-Odasso and Duque, 2005; Cameron et al., 2010; Halfon et al., 2015; Zhou et al., 2016). However, it is somewhat difficult to confirm the cause-effect association between low 25(OH)D levels and frailty because frailty may contribute to the development of vitamin D deficiency because of a reduction in outdoor activity, facilitating a sedentary lifestyle and therefore reducing exposure to sunlight (Artaza-Artabe et al., 2016). Hence, future studies are necessary to establish an optimal serum 25(OH)D concentration in frail elderly persons and the association between vitamin D status and various frailty models (Zhou et al., 2016).

Finally, it should be noted that frailty influences the effects of therapy for various disorders, particularly invasive management in cardiovascular diseases (Singh et al., 2014). Because frail people have a higher risk of complications, conservative or less invasive strategies are preferred, such as percutaneous coronary interventions instead of coronary by-pass grafting for multivessel coronary artery disease (Rumsfeld et al., 1999; Singh et al., 2008). However, even frail patients benefit from current drug therapy although the frailest individuals are less likely to be treated with up-to-date medicines, primarily because of concerns regarding side effects. In studies addressing statin therapy, frail elderly patients with cardiovascular diseases treated with these agents presented a reduced three-year mortality rate irrespective of age and multidimensional impairment (Pilotto et al., 2015, 2016b). In addition, older persons with atrial fibrillation benefit from anticoagulation therapy in terms of lower all-cause death regardless of their functional condition. Nevertheless, there are suggestions that some positive effects of the above treatments may come from better overall care provided to these patients (Pilotto et al., 2016a).

## Aging is in the mind—how to change the mind?

Recently, a concept of “cognitive frailty” has been proposed that is characterized by the coexistence of physical frailty and cognitive impairment (Panza et al., 2006; Kelaiditi et al., 2014). Such an approach is consistent with the multidimensional definition of frailty in which physical, psychological and social factors interplay with one another and consequently potentiate their effects (Figure 1). Cognitive frailty is a heterogeneous clinical syndrome of cognitive impairment (corresponding to the Clinical Dementia Rating Scale [CDR] ≤ 0.5) associated with physical frailty or pre-frailty but independent of dementia resulting from Alzheimer's disease (AD) or other neurodegenerative conditions (Kelaiditi et al., 2014; Panza et al., 2015; Ruan et al., 2015). Two subtypes of cognitive frailty are currently distinguished: reversible and potentially reversible. Reversible cognitive frailty is defined as subjective cognitive decline and/or positive fluid and imaging biomarkers of neurodegeneration or neural injury (CDR < 0.5) that are unrelated to acute medical events or clinical diagnosis of neurodegenerative and mental conditions. Potentially reversible cognitive frailty is identified if physically frail individuals present mild cognitive impairment (CDR = 0.5) (Kelaiditi et al., 2014; Ruan et al., 2015). By combining self-reported symptoms, cognitive tests and biomarkers, one may differentiate (to some extent) cognitive frailty from preclinical AD and other neurological/psychiatric disorders. For this purpose, evidence of the accumulation of amyloid-ß and tau protein within the central nervous system and presence of ApoE ε4 genotype should be considered. Based on different combinations of these biomarkers and the decline of mental function, the cognitive frailty and various preclinical stages of AD may be recognized (Sperling et al., 2013; Ruan et al., 2015). There are a number of studies indicating that physical frailty is a risk factor of cognitive frailty, which in turn is a predictor of overall dementia, particularly vascular dementia (Giannini et al., 2015; Montero-Odasso et al., 2016; Feng et al., 2017). However, precisely how physical frailty or pre-frailty causes cognitive decline is not known (Kelaiditi et al., 2014; Panza et al., 2015; Ruan et al., 2015). It has been shown that cognitive frailty not only predicts dementia but also all-cause mortality among the elderly in both short- and long-term observations (Solfrizzi et al., 2017).

Diverse psychological and social factors may contribute to the development of cognitive impairment. Indeed, aging is associated with an unavoidable awareness of elapsing time, which constantly reminds elderly people of the approaching end of life and significantly affects their psychological state. In addition, a lack of occupation and loneliness may lead to the loss of motivation and purpose. Social programmes that meet only basic demands, such as accommodation and physiological needs, are not sufficient to motivate older people to participate in regular physical, intellectual and social activities. Of note, having a purpose appears to be a key element in encouraging elderly subjects to activate their abilities. It has been shown that volunteer work decreases the risk of becoming frail by 27% after 3 years of follow-up. Providing care for children and paid work may also be associated with some tendency to reduce frailty, however, adjusting for age, disability, and cognitive function attenuates these effects (Jung et al., 2010). An approach integrating a cognitive-behavioral intervention with a faith-based curriculum among a highly sedentary population of older African American women was an effective strategy to increase their physical activity (measured in steps walked per week), which was additionally associated with lowering their blood pressure (Duru et al., 2010). In a large randomized controlled trial, cognitive training conducted in secular community settings improved cognitive abilities over 5 years after the beginning of the intervention (Willis et al., 2006). In another trial, cognitive training designed to enhance attention and information processing and stimulate short-term memory, reasoning and problem-solving abilities significantly decreased frailty. Notably, although the reduction of frailty in the intervention groups was remarkably higher in this study, the control group participants also demonstrated some benefit in terms of diminishing frailty (Ng et al., 2015). The latter suggests that some positive effects may also be achieved only from contact with other people (i.e., the study staff in this case); presumably, providing some attention to and interest in elderly persons may stimulate them toward a more active life.

Social interactions and events may play an important role in keeping life progressing because such circumstances may involve multiple cognitive, social and physical elements and consequently ameliorate various domains of human functioning. One such activity is dancing, which can improve lower body muscle strength and flexibility, aerobic power, static and dynamic balance, agility, and gait speed in older adults and may also enhance lower body bone-mineral content and reduce the incidence of falls and cardiovascular risks (Keogh et al., 2009). Cross-sectional studies provide arguments that elderly people who dance have better balance and gait abilities than aged-matched controls (Verghese, 2006; Zhang et al., 2008). An observational study in older Japanese people revealed that dancing for at least 1year decreased the incidence of falls by 70% (Okubo et al., 2014). However, a recently published randomized controlled trial among residents of retirement villages near Sydney indicated that social dancing programmes did not reduce falls in older adults or cause any significant improvement in cognitive risk factors (Merom et al., 2016). An explanation of these negative results may be that the increase in physical activity in the dance group most likely increases exposure to falls, particularly among elderly individuals who previously experienced such events (Merom et al., 2016). Therefore, a careful investigation of the potential risk of falls should be conducted before elderly subjects are cleared for dancing programmes.

In terms of preventing frailty, the most promising approach appears to be longitudinal community-based strategies integrating multiple dimensions of human activities. The 10-year community-based intervention to prevent frailty in Kusatsu (Japan) effectively improved the functional status of older residents in this area (Shinkai et al., 2016). The project included physical, nutritional and social aspects and targeted enhancing human connections and encouraging participants to continue the group activities after the end of the project. The annual incidence rate for disability decreased, and active life expectancy at age 70 was significantly prolonged. The results of this intervention demonstrate that a community-based approach is a promising technique for preventing or postponing the onset of frailty. Moreover, this was the first study in which regular health check-ups demonstrated having a positive effect on healthy aging in community-dwelling elderly people; the check-ups with comprehensive geriatric assessments were associated with less loss of active life and a reduction in subsequent mortality (Shinkai et al., 2016). The authors also stated that many young residents who worked as survey interviewers became aware of the frailty problem, and older residents became familiar with the concept of healthy aging and accordingly improved their lifestyle (Shinkai et al., 2016).

It appears that broad societies are not aware of frailty as a health problem and that people generally do not realize that they may change their unfavorable trajectory to senility. As populations become older, it is sensible to treat frailty as other population-affecting disorders and implement appropriate preventative measures: adequate information appears to be the first step. Social campaigns should inform societies about age-related frailty and pre-frailty and should also suggest a proper lifestyle to avoid or delay these conditions, as societies do with obesity, diabetes and cardiovascular diseases. The perception of senility requires considerable revision in society and among elderly people themselves. Because we can live longer, it is reasonable to replace the term “aging” with the term “longer life”, which more accurately reflects demographic changes in the upcoming time; such a change in mentality is imperative to properly prepare communities for greater longevity.

The current approach to elderly subjects is routinely directed toward ensuring conditions in which the elderly do not need to keep up with new technologies or lifestyle changes. Although they may feel safer as a result, this approach encourages a type of dependency. In this context, the biggest barrier appears to be new digital technologies that create substantial concern among older people and prevent them from participating in a full social life. As yet, no dedicated programme has addressed this problem, and no systematic approach has been proposed to make elderly persons more self-confident and more familiar with these challenges. Properly devoted studies should address the question of whether such an approach may increase the social and individual activities of elderly people and reduce the prevalence of frailty in the future.

## Conclusions

Early detection of any form of functional degradation in elderly subjects is critical for timely intervention to preserve their biological, psychological and social status. Pre-frailty and frailty are preclinical states, and thus diagnostic instruments should have high potential to capture these conditions in their initial phases to identify persons who may benefit from preventive treatment. Although regular exercise training appears to be the basis for managing pre-frail and frail people, more high-quality trials are needed to determine the most appropriate type of training in older adults. Periodic monitoring of food intake and body weight is essential in elderly people; however, determining the real effectiveness of nutritional supplementations and other probable therapeutic interventions, such as treatment with vitamin D, androgen, antioxidants, and anti-inflammatory agents, requires further investigation to establish their optimal combination and most effective duration. Finally, frailty should be viewed as a population-affecting disease; therefore, a public approach with proper informative strategies regarding frailty itself and concepts of healthy aging should be a key element of global frailty prevention.

## Author contributions

The concept of the article, data collection and analysis: JS and MS. The discussion of the concept and reviewing the data: JS, MS, JaS, ZB, and PF. Drafting the work: JS. Revising the manuscript critically for important intellectual content: JS, MS, JaS, ZB, and PF. Final approval of the version to be published: JS, MS, JaS, ZB, and PF.

### Conflict of interest statement

The authors declare that the research was conducted in the absence of any commercial or financial relationships that could be construed as a potential conflict of interest.
